# Association Between Prescribed Hypnotics in Infants and Toddlers and Later ADHD: A Large Cohort Study from Norway

**DOI:** 10.1007/s10578-020-01039-9

**Published:** 2020-08-09

**Authors:** Ingvild Holdø, Jørgen G. Bramness, Marte Handal, Berit Hjelde Hansen, Vidar Hjellvik, Svetlana Skurtveit

**Affiliations:** 1 Norwegian Centre of Addiction Research, University of Oslo, Oslo, Norway; 2 Norwegian National Advisory Unit on Concurrent Substance Abuse and Mental Health Disorders, Innlandet Hospital Trust, Hamar, Norway; 3 Institute of Clinical Medicine, UiT – Norway’s Arctic University, Tromsö, Norway; 4 Norwegian Institute of Public Health, Skøyen, P.O. Box 222, 0213 Oslo, Norway; 5 Norwegian Center on Expertise for Neurodevelopmental Disorders and Hypersomnias, Oslo, Norway

**Keywords:** ADHD, Sleep disorder, Hypnotics, Infancy

## Abstract

As previously indicated an association may exist between early sleep problems in infants and toddlers, and a diagnosis of attention deficit hyperactivity disorder (ADHD). The aim of this study was to study if this association could be replicated in a complete nationwide cohort of children. Prospective cohort study using national registries. All children born in Norway from January 2004 to December 2010 were included (N = 410,555). Information on hypnotic drugs dispensed to children 0–3 years of age outside of institutions was collected from the Norwegian Prescription Database and used as a proxy for sleep problems. The outcome ADHD (ICD-10), as diagnosed by specialists in the Child Mental Health Service, was obtained from the Norwegian Patient Registry. Data were analysed using weighted estimation in Cox regression. The unadjusted weighted hazard ratio (wHR) for a later diagnosis of ADHD in children dispensed two or more prescriptions for any hypnotic drug, compared to zero prescriptions, was 2.30 [95% confidence interval (CI) 1.63–3.23] for girls and 1.75 (95% CI 1.48–2.07) for boys. For the sedative antihistamine trimeprazine the corresponding wHR was 3.71 (95% CI 1.83–7.52) for girls and 2.78 (95% CI 2.04–3.80) for boys. After adjusting for parental ADHD and parental education the wHR for trimeprazine users was 2.81 (95% CI 1.34–5.88) for girls and 2.33 (95% CI 1.70–3.20) for boys. Infants and toddlers who were dispensed hypnotics had an increased risk of ADHD at school age. This association was most pronounced with the use of trimeprazine, a drug traditionally prescribed to toddlers for sleep problems in Norway. After adjusting for parental ADHD and educational level the risk for ADHD among the trimeprazine users was still more than twice the risk among controls.

## Introduction

Regulation problems such as sleep–wake regulation, crying and transition to solid foods are important challenges for all human babies in the first years of life [1], and problems in these areas often occur together [2, 3]. Longitudinal studies have shown that early regulatory problems are associated with later emotional and behavioural problems [2, 3], a relationship also found when the regulatory problems are early sleep problems [4–7]. Sleep problems also seem to be related to the later development of ADHD symptoms [3, 8–10]. Studies using diagnoses of ADHD as outcome are fewer and results more inconsistent. One study indicated that about one in four children with severe sleep problems qualified for a diagnosis of ADHD at an age of 5.5 years [11], confirmed by a retrospective study where parents of children with a diagnose of ADHD at 8–9 years of age reported persistently elevated levels of sleep problems from infancy [12]. However, a population based longitudinal study among 8253 children found shortened sleep at the age of 5, but not earlier, to be associated with ADHD at the age of 7 years [13]. In a different study sleep problems at the age of 4 years did not predict ADHD diagnosis at the age of 6 when early symptoms of the disorder were adjusted for [14].

Pharmacological treatment of childhood sleep problems is frequent in many countries [15, 16]. An Australian study found that among parents of children 0–24 months, 6% had received an over-the-counter (OTC) medicine to induce sleep in the child [17]. In Norway there is a long standing clinical tradition of prescribing the antihistamine trimeprazine for infants and toddlers with sleep problems [18, 19], even if there have been some concern about the effect and safety of the drug in this age group [20–22]. The use of trimeprazine has declined over the last 10 years [23, 24]. Some 3% of the children born in 2008 filled a prescription for the drug before 3 years of age and the peak incidence of trimeprazine prescriptions at age 18 months [25].

Norway has several national health registries with compulsory and automatically collected information. Several registries can be linked on an individual level, making it possible to conduct large cohort studies, with information from different sources and follow up over time. In such national registries, only emigrants are lost to follow up. We wanted to utilize these registries to create a large-scale cohort and study if previous findings on a relationship between early sleep problems and later development of ADHD could be replicated. As ICD-10 diagnosis for sleep disturbances are rarely used in the paediatric population, dispensed hypnotic medications for children was used as the exposure. We studied the association between dispensed hypnotics before age of 3 years, and diagnosed ADHD between 5 and 11 years of age, in all children born in Norway between 2004 and 2010. We further wanted to explore to what extent adjusting for parental ADHD and parental educational level affected the association.

## Methods

### Data Sources

In this cohort study, we linked information from the Norwegian Prescription Database (NorPD), the Norwegian Patient Registry (NPR) and Statistics Norway, and used data spanning an 12 year time period (2004–2015). The unique person identifier held by all Norwegian inhabitants enabled us to link the different registries at an individual level.

All pharmacies have, since 2004, been obliged to register electronically all dispensed prescribed drugs to individuals and send the information to the NorPD [26]. Each prescription is registered with drug information, including Anatomical Therapeutic Chemical (ATC) code [27, 28], date of dispensing and patient identifiers. The NorPD provided information on dispensed hypnotic drugs and stimulant medication*.*

The NPR is an administrative database containing activity data from all hospitals, private specialists and outpatient clinics in Norway. Reporting to the NPR is mandatory and linked to the governmental reimbursement system for funding of health services. Every admission to a hospital or appointment with a specialist or outpatient clinic is registered with information such as date, diagnosis and patient data. All Norwegian residents are covered by the public health care system through a national insurance scheme. Health care is free of charge for children under 16 years. This implies that our data should capture all children diagnosed with ADHD in Norway. All diagnoses are reported according to the International Classification of Diseases, 10th Revision, ICD-10 [29]. Individual data are available in the NPR since January 1st 2008, and we have used data from 2008 to 2015.

Statistics Norway provided the link between parents and children and information on the highest achieved educational level in 2014. Education is reported in nine categories according to the Norwegian Standard Classification of Education [30]. Dates of death and emigration were available from the National Registry.

### Population/Data Selection

Data from the NorPD, the NPR, Statistics Norway and the National Registry were linked using the encrypted personal identifier. All children born in Norway in the years 2004–2010 were initially included in our study population, in total 429,158 children. 18,603 Children who died or emigrated before the age of 5 years were excluded. Our final selection consisted of 410,555 children.

### Exposure

From the NorPD, we collected information about all prescriptions for children under 3 years, for drugs known to be used in Norway as hypnotic drugs. In addition to the traditional anxiolytic and hypnotic drugs we also chose to include first generation antihistamines classified in ATC under respiratory system—antihistamines for systemic use (R06), but known to be sedating. There is a clinical tradition for using sedating antihistamines for sleep induction in small children in Norway [19, 31]. These three medications were trimeprazine (R06AD01), prometazine (R06AD02), and dexchlorpheniramine (R06AB02). Hypnotics were defined as: anxiolytics (ATC Code N05BA: benzodiazepines), hypnotics and sedatives (N05C: benzodiazepine hypnotics, z-hypnotics, melatonin and chloral hydrate), trimeprazine (R06AD01), prometazine (R06AD02), and dexchlorpheniramine (R06AB02). All of these medications were only available by prescription at the time and not over-the-counter sale. Which means that all medication in this category should be captured by the NorPD [32].

As we only had access to birth month and birth year, we assigned a birth date in order to use the age of the children in the analysis. We chose to set the birth date to the 15th of the month for all children. This over- or under-estimated the actual birthdate by no more than 16 days. We wanted to investigate use of medication in infancy and toddler age, which is commonly considered to be from birth until 36 months of age. From the estimated birth date, a 36 months “exposure window” was calculated and we collected all dispensed prescriptions in this period. The medications were analysed separately and grouped together as the variable “all hypnotics”. A negligible number of toddlers received more than one hypnotic drugs during the exposure window. Also, investigating the effect of combined medication use was beyond the scope of this study. We stratified the exposure variable into those receiving no prescriptions, one prescription and two or more prescriptions. From before we know that around 70% of children who receive trimeprazine before 3 years of age only receive one prescription [25].

### Outcome

For outcome, we identified all children who had at least two registered visits in specialised health care, resulting in an ICD-10 F90 (hyperkinetic disorder/ADHD) diagnosis from around school age of 5 years and onwards, either as main or secondary diagnosis in the NPR in the years 2008–2015. Time of outcome was for these children defined as time of the first diagnosis. Although the ICD-10-diagnosis F90 hyperkinetic disorder is slightly narrower than the DSM-IV diagnosis ADHD it is considered equivalent in this study. The children were censored at death, at emigration or as of December 31st 2015, whichever occurred first.

### Confounding Factors

For confounding factors, parental education was included to control for socioeconomic status of the family. Education was stratified into two levels; “low” up to high school diploma and “high” including college and university education. The level of education for one or both parents was registered for 407,608 children. All parents who had ever (2008–2015) been diagnosed with F90 hyperkinetic disorder registered in NPR or had ever (2004–2015) been dispensed a prescription for a stimulant medication (amphetamine N06BA01, dexamphetamine N06BA02, methylphenidate N06BA04, atomoxetine N06BA09, lisdexamphetamine N06BA12) in NorPD were considered to have ADHD. Treatment with stimulant medication is regulated by the Norwegian authorities and must be initiated by a specialist in psychiatry, neurology or paediatrics.

### Statistical Analysis

Two types of statistical analyses were performed; χ^2^ testing and weighted Cox regression.

A bivariate analysis with χ^2^ test was performed for the association of children’s ADHD with use of (i) any hypnotic drug, (ii) dexchlorpheniramine (the most prevalent drug) and (iii) trimeprazine (the second most prevalent drug). We performed the same analysis for (iv) parental ADHD and (v) parental educational level. Cramér’s bias corrected V was computed using the “cramerV” function in the R-package “rcompanion”.

Since follow-up time varied from 1 day to 7 years, Cox regression analyses were performed to estimate hazard rate ratios (HRs) associated with use of (i) any hypnotic drug, (ii) dexchlorpheniramine and (iii) trimeprazine. Time of outcome was the time of the first of two ADHD diagnoses, and exposure was a 3-level categorical variable: 0 (reference), 1, or ≥ 2 prescriptions before age 3. The Cox regression was adjusted with inclusion of parental ADHD as a two- level categorical variable; no parental ADHD (reference)/at least one parent with ADHD, and maternal and paternal education as two two-level categorical variables; high school or less/university or college education (reference). We present Kaplan–Meier survival plots, and we tested the proportional hazards assumption using the “cox.zph” function in R. In some cases there was a significant deviance from proportionality at a 5% level, and we therefore performed a weighted Cox regressions [33] using the “coxphw” function in R as well. Finally, we tested for interaction between gender and exposure.

The analyses were performed using IBM SPSS statistics version 22 and R version 3.5.2.

## Results

Before the age of 3 years, 10.4% of the girls and 12.3% of the boys had been dispensed at least one prescription of a hypnotic drug. The two most prevalent drugs were dexchlorpheniramine (girls 6.7% and boys 7.9%) and trimeprazine (2.8% and 3.6%). The prevalence of the other hypnotics was as follows: diazepam (girls 1.5%, boys 1.7%), melatonin (0.1% and 0.2%), nitrazepam (0.05% and 0.06%), midazolam (0.02% and 0.03%), clobazam (0.02% and 0.02%), oxazepam, promethazine, zopiclone, zolpidem (all < 0.01%) (data not shown in table).

Use of hypnotic drugs at age 0–3 was more prevalent among children who later received an ADHD diagnosis than among other children (girls 18.9% vs 10.3%, boys 20.1% vs 12.2%, Table 1). For trimeprazine the corresponding numbers were 7.6% vs 2.8% in girls and 7.4% vs 3.5% in boys (Table 1).

**Table 1:** Bivariate analysis showing the association between dispensed hypnotic drugs to girls and boys aged 0–3 years and ADHD diagnosis after the age of 5 years

	Girls								Boys							
	No ADHD		ADHD		N total	χ^2a^	p-value^a^	V^b^	No ADHD		ADHD		N total	χ^2a^	p-value^a^	V^b^
All hypnotics^c^																
No	177,845	89.7%	1344	81.1%	179,189	129	< 0.001	0.025	180,770	87.8%	3845	79.9%	184,615	274	< 0.001	0.036
Yes	20,451	10.3%	313	18.9%	20,764^d^				25,019	12.2%	968	20.1%	25,987^e^			
Dexchlorpheniramine																
No	185,036	93.3%	1490	89.9%	186,526	30	< 0.001	0.012	189,725	92.2%	4237	88.0%	193,962	111	< 0.001	0.023
Yes	13,260	6.7%	167	10.1%	13,427^d^				16,064	7.8%	576	12.0%	16,640^e^			
Trimeprazine																
No	192,819	97.2%	1531	92.4%	194,350	140	< 0.001	.026	198,621	96.5%	4455	92.6%	203,076	212	< 0.001	0.032
Yes	5477	2.8%	126	7.6%	5603^d^				7168	3.5%	358	7.4%	7526^e^			

^a^Test observator and p-value from χ^2^ test with 1 degree of freedom

^b^Cramér’s bias-corrected V

^c^Hypnotic drugs include trimeprazine, promethazine, dexchlorpheniramine, zolpidem, zopiclone, melatonin, midazolam, clobazam, nitrazepam and oxazepam

^d^Number of girls who received 2 or more prescriptions was; for all hypnotics 5573, for dexchlorpheniramine 3010 and for trimeprazine 1496

^e^Number of boys who received 2 or more prescriptions was; for all hypnotics 7992, for dexchlorpheniramine 4445 and for trimeprazine 2195

Of the children who developed ADHD, 17.4% had at least one parent with ADHD whereas only 2.6% of the children without ADHD had at least one parent with ADHD (Table 2). Among the children with and without ADHD the percentage of maternal lower education was 66.3% and 46.8%, respectively (Table 2).

**Table 2:** Bivariate analysis showing parents’ ADHD and parents’ education by whether or not the child was diagnosed with ADHD after 5 years of age

	ADHD, child				N total	χ^2^ (df)^c^	p-value^c^	V^d^
	No		Yes					
Mother ADHD								
No	398,598	98.6%	5748	88.8%	404,346	4101 (1)	< 0.001	0.010
Yes	5487	1.4%	722	11.2%	6209			
Father ADHD								
No	398,653	98.7%	5985	92.5%	404,638	1692 (1)	< 0.001	0.006
Yes	5432	1.3%	485	7.5%	5917			
One or both parents have ADHD								
No	393,680	97.4%	5345	82.6%	399,025	5114 (1)	< 0.001	0.011
Yes	10,405	2.6%	1125	17.4%	11,530			
Mother’s education^a^								
NA^b^	2931	0.7%	16	0.3%	2947	968 (2)	< 0.001	0.005
Low	189,267	46.8%	4287	66.3%	193,554			
High	211,887	52.4%	2167	33.5%	214,054			
Father's education^a^								
NA^b^	2931	0.7%	16	0.3%	2947	925 (2)	< 0.001	0.005
Low	246,381	61.0%	5145	79.5%	251,526			
High	154,773	38.3%	1309	20.2%	156,082			

^a^Missing values were replaced by information from the other parent. For 2947 children none of the parents had registered education information

^b^Information not available (NA) for any parent

^c^Test observator and [degrees of freedom (df)] and p-value from χ^2^ test

^d^Cramér’s bias corrected V

**Fig. 1:**
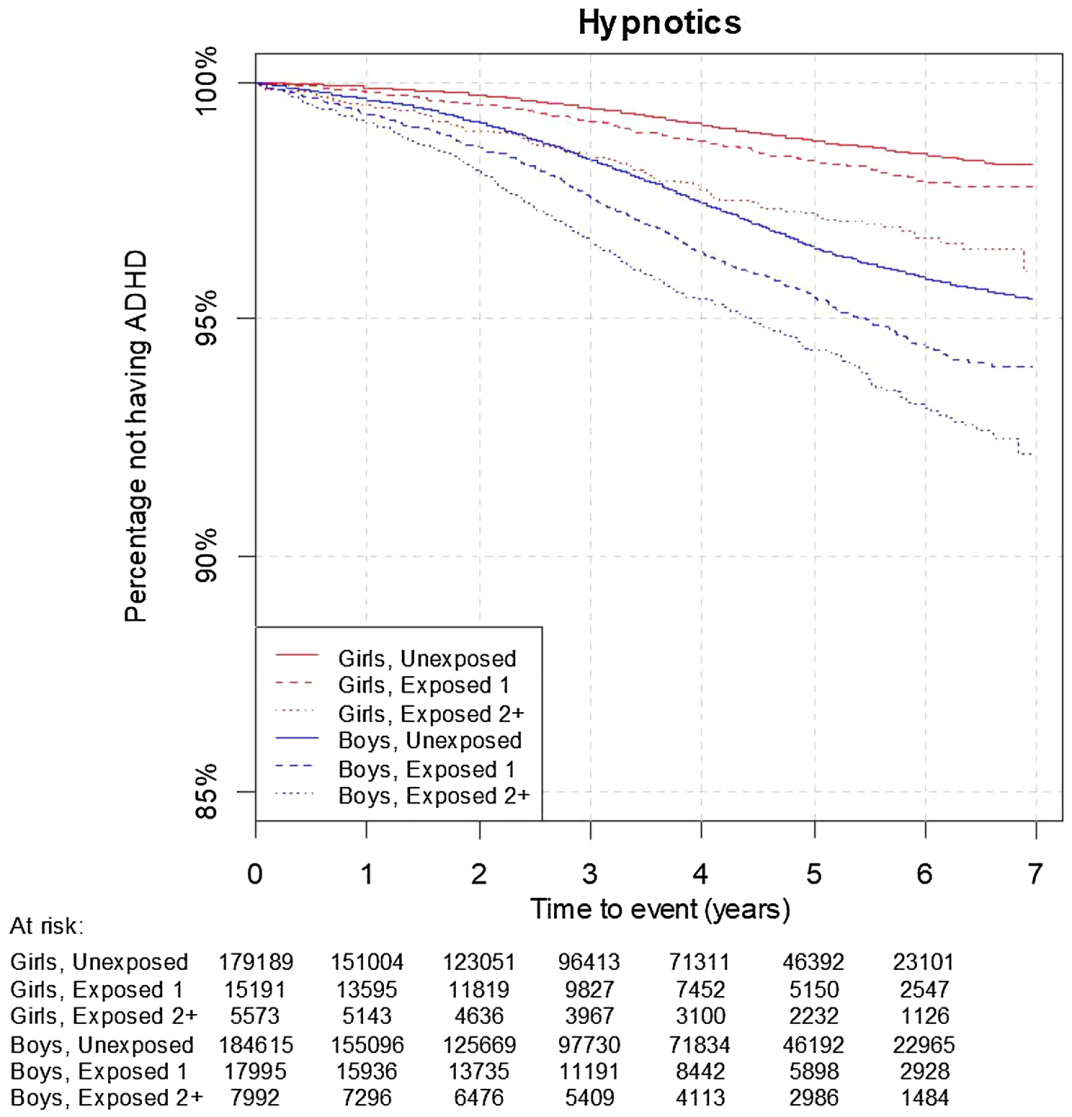
Kaplan–Meier survival plots for hypnotics. The unexposed, exposed 1 and exposed 2+ group had 0, 1, and 2+ prescriptions of any hypnotic, respectively at age 0–3 years. Follow-up started at age 5. Numbers in the at risk table below the figure show the population at risk at baseline and after 1, 2,…,6 years

**Fig. 2:**
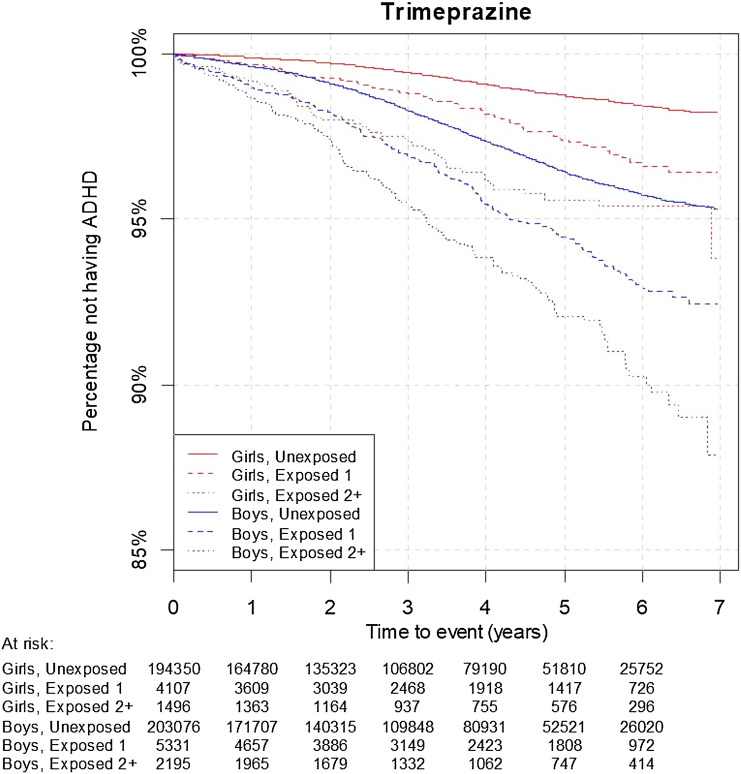
Kaplan–Meier survival plots for trimeprazine. The unexposed, exposed 1 and exposed 2+ group had 0, 1, and 2+ prescriptions of trimeprazine, respectively at age 0–3 years. Follow-up started at age 5. Numbers in the at risk table below the figure shows at risk population at baseline and after 1, 2,…,6 years

The proportional hazards assumption of the Cox regression was not fulfilled for all regressions. For girls, the HR associated with ≥ 2 prescriptions tended to decrease over time both for hypnotics and for trimeprazine (p-values for proportionality were 0.001 and 0.0005, respectively). This can also be seen from the Kaplan–Meier survival plots (Figs. 1, 2). In particular for trimeprazine, the survival curves for exposed with ≥ 2 prescriptions start to drop before the curves for the unexposed. The p-value for proportionality was < 0.05 for 4 of the 12 adjusted HRs in Table 3. We therefore present the average the HRs from the weighted Cox regression (wHR) in Table 3 for all HRs.

**Table 3:** The hazard ratio of ADHD at school age after using hypnotic drug trimeprazine between the ages 0 and 3 years for children born between 2004 and 2011

	Unadjusted			Adjusted^a^		
	HR	95% CI	p-value^b^	HR	95% CI	p-value^b^
Girls						
No hypnotics (reference)	1.00			1.00		
All hypnotics one prescription	1.23	1.02–1.49	0.026	1.18	0.98–1.42	0.080
All hypnotics twice or more	2.30	1.63–3.23	< 0.001	1.99	1.40–2.83	< 0.001
No dexchlorpheniramine (reference)	1.00			1.00		
Dexchlorpheniramine one prescription	1.31	0.94–1.82	0.113	1.27	0.91–1.78	0.161
Dexchlorpheniramine twice or more	1.23	0.85–1.80	0.270	1.11	0.76–1.62	0.587
No trimeprazine (reference)	1.00			1.00		
Trimeprazine one prescription (ref 0)	2.02	1.52–2.69	< 0.001	1.76	1.32–2.34	< 0.001
Trimeprazine twice or more	3.71	1.83–7.52	< 0.001	2.81	1.34–5.88	0.006
Boys						
No hypnotics (reference)	1.00			1.00		
All hypnotics one prescription (ref 0)	1.32	1.18–1.47	< 0.001	1.28	1.15–1.42	< 0.001
All hypnotics twice or more	1.75	1.48–2.07	< 0.001	1.58	1.34–1.87	< 0.001
No dexchlorpheniramine (reference)	1.00			1.00		
Dexchlorpheniramine one prescription (ref 0)	1.27	1.09–1.48	0.002	1.24	1.07–1.44	0.005
Dexchlorpheniramine twice or more	1.27	1.04–1.55	0.018	1.18	0.97–1.44	0.100
No trimeprazine (reference)	1.00			1.00		
Trimeprazine one prescription (ref 0)	1.62	1.36–1.93	< 0.001	1.50	1.25–1.78	< 0.001
Trimeprazine twice or more	2.78	2.04–3.80	< 0.001	2.33	1.70–3.20	< 0.001

^a^The full model is adjusted for paternal and maternal education (high/low) and for parental ADHD (no/at least one parents with ADHD)

^b^With a Bonferroni correction for multiple testing a test would have to have p < 0.0042 to be significant

With standard Cox regression we also found significant gender interactions, with a higher HR of ADHD associated with ≥ 2 prescriptions both for all hypnotics and for trimeprazine in girls than in boys (p-values for interaction were 0.005 and 0.021 for all hypnotics and for trimeprazine, respectively). The weighted Cox regression did not reveal any statistically significant interactions, but we have chosen to still present the results separately for boys and girls.

The unadjusted wHR for receiving a later diagnosis of ADHD when dispensed one prescription for any hypnotics from birth to 3 years of age, as compared to no prescriptions, was 1.23 (95% CI 1.02–1.49) for girls and 1.32 for boys (1.18–1.47) (Table 3). The wHR when being dispensed at least two prescriptions as compared to no prescriptions was 2.30 (1.63–3.23) for girls and 1.75 (1.48–2.07) for boys (Table 3). Being dispensed dexchlorpheniramine was less related to a future ADHD diagnosis. For those who filled one prescriptions for trimeprazine the wHR was 2.02 (1.52–2.69) for girls and 1.62 (1.36–1.93) for boys and for two or more prescriptions the figures were 3.71 (1.83–7.52) and 2.78 (2.04–3.80) for girls and boys respectively. Parent’s educational level and especially a parental ADHD diagnosis was highly related to the child’s later ADHD diagnosis (Table 2). After adjusting for the important factors (parent’s ADHD and parent’s educational level) children who received two or more prescriptions for any hypnotic drug had a HR for being diagnosed with ADHD of 1.99 (1.40–2.83) for girls and 1.58 (1.34–1.87) for boys. The adjusted HR of ADHD after being dispensed trimeprazine once was 1.76 (1.32–2.34) for girls and 1.50 (1.25–1.78) for boys. After being dispensed trimeprazine twice or more, the HR was 2.81 (1.34–5.88) for girls and 2.33 (1.70–3.20) for boys, respectively.

## Discussion

Being dispensed any hypnotic drug twice or more before the age of 3 years, doubled the risk of a later ADHD diagnosis for girls and increased the risk by 60% for boys even after controlling for known risk factors such as parental ADHD and parental educational level. The risk after being dispensed the sedative antihistamine trimeprazine was even higher. The increased risk of ADHD diagnosis when receiving two or more prescriptions as opposed to one prescription indicates a dose–response relationship where more severe sleep problems further increased the risk of later receiving a diagnosis of ADHD.

From birth to the age of 3 years, a little over 10% of both girls and boys received prescriptions for sleep inducing drugs. The most frequent drugs, dexchlorpheniramine and trimeprazine, are both first generation antihistamines, not traditional hypnotics. But unlike newer antihistamines these substances cross the blood–brain barrier and have sedating effects. In Norway there is a long-standing clinical tradition for prescribing the sedative antihistamine trimeprazine on a large scale for infants and toddlers as a sleep inducer [24]. Dexchlorpheniramine and trimeprazine have similar effects, but the reimbursements (unpublished data). Among children 0–3 years of age more than 40% of prescriptions for dexchlorpheniramine were reimbursed for allergy whereas only 2% of alimemazine prescriptions were reimbursed for allergy/pruritus. As sleep problems is the only other indication for prescribing trimeprazine this could mean that trimeprazine to a large extent is used to treat sleep disturbances. Recommending antihistamines for sleep induction is well known in US settings [34] and studies from Europe report frequent use of systemic antihistamines for small children [35, 36]. Melatonin has over the last years emerged as the first choice hypnotic for paediatric insomnia [37] although long term safety studies on children are still missing [38]. Melatonin is not predominantly prescribed for the youngest age group in Norway [18, 23], and is mainly prescribed for children with psychiatric or neurological conditions [37]. In the observational time of the study melatonin was not available for sale in Norway without prescription. In Norway, it is thus reasonable to use prescriptions for trimeprazine as a proxy for challenges with sleep in infants and toddlers.

Girls who received sleep medication were more likely to be diagnosed with ADHD than boys who received sleep medication. Several studies find that toddler boys sleep less that girls [39, 40]. Perhaps is the threshold for sleep medication in boys lower than that for girls, and the girls who received a prescription for a sleep medication had more serious sleep problems than boys.

The association between dispensed hypnotic drugs and later ADHD could have several explanations. Although we believe that hypnotic use serves as a proxy for an underlying sleep problem, it could be argued that a direct drug effect on the brain development explains the association. The aetiology of ADHD is multifaceted, but even if drugs like trimeprazine and dexchlorpheniramine influence a number of receptors in the brain [41], such a direct effect on the brain facilitating ADHD seems biologically unlikely; mostly because the drugs are used for a short period of time [25]. Also, the differences in risk following the quite similar drugs trimeprazine and dexchlorpheniramine [42–44] used for different indications, trimeprazine used as hypnotic and dexchlorpheniramine for allergy, suggest that the increase in risk of ADHD is not a drug effect but could, rather, be related to the reasons for the drug use.

Problems with sleep are frequent in both adults and children with ADHD [45, 46]. A meta-analysis has shown that infant sleep problems predict difficult temperament or problem behaviour later in life [3], but the analysis was based on small and heterogeneous studies mainly based on maternal report of regulation problems. Most studies used the parent report Child Behaviour Checklist as measurement of behavioural problems. Several studies have shown that sleep problems are related to the later development of ADHD symptoms [3, 8–10]. Studies using diagnostic ADHD show both an increase in ADHD related to early severe sleeping problems [11, 12], while other fail to replicate this [13, 14]. The present study found an association between dispensed hypnotics from birth to age 3 and a diagnosis of ADHD in school age set by specialists in Child and Adolescent Psychiatry. Using dispensed hypnotic drugs as a proxy for severe sleep problems, our cohort study supports those who have found a relationship between early sleeping problems and later diagnosis of ADHD in a population of more than 400,000 children followed from the age of 5 years up to 11 years, without loss to follow up [11, 47].

There could be several explanations for the association between sleep problems and later ADHD. Sleep problems could be an early symptom of ADHD preceding diagnosis. Other studies have shown modest to strong stability of ADHD-like symptoms from 18 months and throughout preschool years [48]. Alternatively, early sleep problems and ADHD could have shared aetiology. Dysregulation of the hypothalamic–pituitary–adrenocortical axis or circadian rhythm disturbances could affect both sleep regulation and attention or activity levels [49, 50]. There is also evidence pointing to a common factor of abnormalities in the dopamine system [51]. Lastly, sleep problems could be causally linked to ADHD. Several studies describe how limited amounts of sleep can cause impairment in neurobehavioral functioning [52, 53]. Sleep deprivation might influence the development of ADHD, either directly via neural loss or indirectly in a cascade where individuals who experience loss of sleep are more susceptible to negative stimuli and show more negative affect [52–54].

The association between dispensed sleep medication and later ADHD diagnosis could be biased by family and social background. Family adversities have been shown to be a more important explanation for the development of hyperkinetic traits than infant regulation problems [55]. The association between dispensed sleep medication and ADHD in childhood is also confounded by parental ADHD. Parents with ADHD could, through their own sleep problems, have low tolerance for small children’s variable sleep patterns and be more likely to seek medical advice and have sleep medications prescribed for their children. A previous study showed that hypnotics dispensed to parents doubled the chance of a prescription for trimeprazine being filled for a toddler [25]. However, in our study, use of hypnotic drugs was associated with an increased risk of ADHD independently of parental ADHD and parental education.

The ADHD prevalence rates in the present study were low compared to what is found internationally [56], but similar to other studies from Scandinavia [57, 58]. The use of ICD-10 criteria for diagnosis could explain some of this discrepancy [59]. In addition, the prevalence of ADHD increases steeply during childhood [58, 60] and we may have underestimated the prevalence of ADHD due to a limited follow-up period. It is, however, unlikely that the timing of diagnosis is related to the use of hypnotic drugs, and thus our low prevalence rates will not bias the calculated risk.

We do unfortunately not have information on sleep diagnosis in the age 0–3 years as the Norwegian prescription database does not contain diagnosis unless medications are reimbursed and hypnotics are not reimbursed for insomnia or sleep disturbances in general [32]. Sleep diagnosis were also not available from the Norwegian Patient Registry as there seems to be a clinical tradition for not using the ICD-10 G47 Sleep Disorders diagnosis for children. Using sleep medications as a proxy for sleep problems is an effort to collect large objective data for a complex condition for which data on diagnosis are not available.

ICD-10 diagnosis of hyperkinetic disorder is narrower than the DSM IV diagnosis of ADHD, as the inattentive type ADHD is not formally included in ICD-10. This subgroup was thus not included in our study. This could introduce an unknown bias, but overall, the exposed group in this study probably includes children with more severe sleep problems and possibly more severe forms of ADHD, possibly leading to a slight overestimation of the association between sleep problems and ADHD.

The proportional hazards assumption of the Cox regression was not fulfilled for all of the regressions, and we therefore present average hazard ratios from weighted estimation in Cox regression. These were quite close to the estimates from standard Cox regression although the confidence intervals were roughly twice as large.

## Summary

This study investigates a complete cohort of 410,555 children born in Norway in the years 2004–2010. We have captured dispensed hypnotic drugs from birth to 3 years of age and all diagnoses for ADHD from 5 years of age and up to the age of 11 years set by specialists in Child and Adolescent Psychiatry. It has previously been shown that infant regulation problems, including sleep problems, is associated with ADHD diagnosis. We replicate this finding in large cohort, where continuous data collection ensures no recall bias and no loss to follow up. We find that the use of hypnotic drugs before 3 years of age signifying substantial sleeping problems increases the risk of a later ADHD diagnosis. This was especially true for the antihistaminergic drug, trimeprazine.

## Data Availability

The data are not available for other researchers as such, only after performing the same procedure of applying, getting permissions and funding the costs of data. Only public registry data were used in this publication.

## Abbreviations

ADHD: Attention deficit hyperkinetic disorder
NorPD: Norwegian Prescription Database
NPR: Norwegian Patient Registry
HR: Hazard ratio

## References

[1] Wolff PH (1987). The development of behavioral states and the expression of emotions in early infancy.

[2] Bilgin A, Baumann N, Jaekel J, Breeman LD, Bartmann P, Bauml JG, Avram M, Sorg C, Wolke D (2018). Early crying, sleeping, and feeding problems and trajectories of attention problems from childhood to adulthood. Child Dev.

[3] Hemmi MH, Wolke D, Schneider S (2011). Associations between problems with crying, sleeping and/or feeding in infancy and long-term behavioural outcomes in childhood: a meta-analysis. Arch Dis Child.

[4] Sivertsen B, Harvey AG, Reichborn-Kjennerud T, Torgersen L, Ystrom E, Hysing M (2015). Later emotional and behavioral problems associated with sleep problems in toddlers: a longitudinal study. JAMA Pediatr.

[5] Sadeh A, Tikotzky L, Kahn M (2014). Sleep in infancy and childhood: implications for emotional and behavioral difficulties in adolescence and beyond. Curr Opin Psychiatry.

[6] Touchette E, Cote SM, Petit D, Liu X, Boivin M, Falissard B, Tremblay RE, Montplaisir JY (2009). Short nighttime sleep-duration and hyperactivity trajectories in early childhood. Pediatrics.

[7] Winsper C, Wolke D (2014). Infant and toddler crying, sleeping and feeding problems and trajectories of dysregulated behavior across childhood. J Abnorm Child Psychol.

[8] Armstrong JM, Ruttle PL, Klein MH, Essex MJ, Benca RM (2014). Associations of child insomnia, sleep movement, and their persistence with mental health symptoms in childhood and adolescence. Sleep.

[9] Kidwell KM, Hankey M, Nelson JM, Espy KA, Nelson TD (2017). Child executive control as a moderator of the longitudinal association between sleep problems and subsequent attention-deficit/hyperactivity disorder symptoms. J Pediatr Psychol.

[10] Gregory AM, Eley TC, O'Connor TG, Plomin R (2004). Etiologies of associations between childhood sleep and behavioral problems in a large twin sample. J Am Acad Child Adolesc Psychiatry.

[11] Thunstrom M (2002). Severe sleep problems in infancy associated with subsequent development of attention-deficit/hyperactivity disorder at 5.5 years of age. Acta Paediatr.

[12] Williams KE, Sciberras E (2016). Sleep and self-regulation from birth to 7 years: a retrospective study of children with and without attention-deficit hyperactivity disorder at 8 to 9 years. J Dev Behav Pediatr.

[13] Scott N, Blair PS, Emond AM, Fleming PJ, Humphreys JS, Henderson J, Gringras P (2013). Sleep patterns in children with ADHD: a population-based cohort study from birth to 11 years. J Sleep Res.

[14] Steinsbekk S, Wichstrom L (2015). Stability of sleep disorders from preschool to first grade and their bidirectional relationship with psychiatric symptoms. J Dev Behav Pediatr.

[15] Stojanovski SD, Rasu RS, Balkrishnan R, Nahata MC (2007). Trends in medication prescribing for pediatric sleep difficulties in US outpatient settings. Sleep.

[16] Heussler H, Chan P, Price AM, Waters K, Davey MJ, Hiscock H (2013). Pharmacological and non-pharmacological management of sleep disturbance in children: an Australian Paediatric Research Network survey. Sleep Med.

[17] Trajanovska M, Manias E, Cranswick N, Johnston L (2010). Use of over-the-counter medicines for young children in Australia. J Paediatr Child Health.

[18] Hartz I, Furu K, Bratlid T, Handal M, Skurtveit S (2012). Hypnotic drug use among 0–17 year olds during 2004–2011: a nationwide prescription database study. Scand J Public Health.

[19] Sanz EJ (1998). Drug prescribing for children in general practice. Acta Paediatr.

[20] Sponheim S, Aune H, Gulliksen M, Morland J (1990). Pharmacokinetics of trimeprazine in children. Pharmacol Toxicol.

[21] Kahn A, Hasaerts D, Blum D (1985). Phenothiazine-induced sleep apneas in normal infants. Pediatrics.

[22] Mann NP (1981). Trimeprazine and respiratory depression. Arch Dis Child.

[23] Norwegian Institute of Public (2018) In: Health NIoP (ed) Norwegian Prescription Database (NorPD), Reseptregisteret. Norwegian Institute of Public Health. https://www.norpd.no. Accessed 1 May 2020

[24] Straand J, Rokstad K, Heggedal U (1998). Drug prescribing for children in general practice. A report from the More and Romsdal Prescription Study. Acta Paediatr.

[25] Holdo I, Handal M, Skurtveit S, Bramness JG (2013). Association between prescribing hypnotics for parents and children in Norway. Arch Dis Child.

[26] Furu K (2008). Establishment of the nationwide Norwegian prescription database (NorPD)—new opportunities for research in pharmacoepidemiology in Norway. Nor Epidemiol.

[27] WHO Collaborating Centre for Drug Statistics Methodology (2015). Guidelines for ATC Classification and DDD Assignment.

[28] WHO Collaborating Centre for Drug Statistics Methodology (2005). Guidelines for ATC Classification and DDD Assignment.

[29] WHO (1993). The ICD-10 classification of mental and behavioural disorders: diagnostic criteria for research.

[30] Statistics Norway (2000). Norwegian Standard Classification of Education.

[31] Slordal L, Bramness JG (2008). Is alimemazine a suitable sleeping agent for children?. Tidsskr Den Nor Legeforening.

[32] Norwegian Pharmaceutical Product Compendium—Vallergan. Sanofi-Aventis. https://www.felleskatalogen.no/medisin/vallergan-sanofi-aventis-564981. Accessed 1 May 2020

[33] Schemper M, Wakounig S, Heinze G (2009). The estimation of average hazard ratios by weighted Cox regression. Stat Med.

[34] Owens JA, Rosen CL, Mindell JA (2003). Medication use in the treatment of pediatric insomnia: results of a survey of community-based pediatricians. Pediatrics.

[35] Benard-Laribiere A, Jove J, Lassalle R, Robinson P, Droz-Perroteau C, Noize P (2015). Drug use in French children: a population-based study. Arch Dis Child.

[36] de Vries TW, van Hunsel F (2016). Adverse drug reactions of systemic antihistamines in children in the Netherlands. Arch Dis Child.

[37] Hartz I, Handal M, Tverdal A, Skurtveit S (2015). Paediatric off-label use of melatonin—a register linkage study between the Norwegian Prescription Database and Patient Register. Basic Clin Pharmacol Toxicol.

[38] Andersen LP, Gögenur I, Rosenberg J, Reiter RJ (2016). The safety of melatonin in humans. Clin Drug Investig.

[39] McDonald L, Wardle J, Llewellyn CH, van Jaarsveld CH, Fisher A (2014). Predictors of shorter sleep in early childhood. Sleep Med.

[40] Blair PS, Humphreys JS, Gringras P, Taheri S, Scott N, Emond A, Henderson J, Fleming PJ (2012). Childhood sleep duration and associated demographic characteristics in an English cohort. Sleep.

[41] Goodman LS, Brunton LL, Chabner B, Knollmann BrC (2011) Goodman and Gilman’s pharmacological basis of therapeutics. In: Brunton LL (ed), Chabner BA, Knollmann BC (associate editors) Goodman and Gilman’s pharmacological basis of therapeutics, 12th edn. McGraw-Hill, New York

[42] Poluzzi E, Raschi E, Godman B, Koci A, Moretti U, Kalaba M, Wettermark B, Sturkenboom M, De Ponti F (2015). Pro-arrhythmic potential of oral antihistamines (H1): combining adverse event reports with drug utilization data across Europe. PLoS ONE.

[43] von Maur K (1985). Antihistamine selection in patients with allergic rhinitis. Ann Allergy.

[44] Herman SM, Vender RB (2003). Antihistamines in the treatment of dermatitis. J Cutan Med Surg.

[45] Wang B, Isensee C, Becker A, Wong J, Eastwood PR, Huang RC, Runions KC, Stewart RM, Meyer T, Bruni LG (1874). Developmental trajectories of sleep problems from childhood to adolescence both predict and are predicted by emotional and behavioral problems. Front Psychol.

[46] Bjorvatn B, Brevik EJ, Lundervold AJ, Halmoy A, Posserud MB, Instanes JT, Haavik J (2017). Adults with attention deficit hyperactivity disorder report high symptom levels of troubled sleep, restless legs, and cataplexy. Front Psychol.

[47] Gurevitz M, Geva R, Varon M, Leitner Y (2014). Early markers in infants and toddlers for development of ADHD. J Atten Disord.

[48] Overgaard KR, Aase H, Torgersen S, Reichborn-Kjennerud T, Orbeck B, Myhre AM, Zeiner P (2014). Continuity in features of anxiety and attention deficit/hyperactivity disorder in young preschool children. Eur Child Adolesc Psychiatry.

[49] Ma L, Chen YH, Chen H, Liu YY, Wang YX (2011). The function of hypothalamus–pituitary–-adrenal axis in children with ADHD. Brain Res.

[50] Pesonen AK, Kajantie E, Heinonen K, Pyhala R, Lahti J, Jones A, Matthews KA, Eriksson JG, Strandberg T, Raikkonen K (2012). Sex-specific associations between sleep problems and hypothalamic–pituitary–adrenocortical axis activity in children. Psychoneuroendocrinology.

[51] Yoon SY, Jain U, Shapiro C (2012). Sleep in attention-deficit/hyperactivity disorder in children and adults: past, present, and future. Sleep Med Rev.

[52] Um YH, Hong SC, Jeong JH (2017). Sleep problems as predictors in attention-deficit hyperactivity disorder: causal mechanisms, consequences and treatment. Clin Psychopharmacol Neurosci Off Sci J Korean Coll Neuropsychopharmacol.

[53] Hvolby A (2015). Associations of sleep disturbance with ADHD: implications for treatment. Atten Deficit Hyperact Disord.

[54] Williams KE, Berthelsen D, Walker S, Nicholson JM (2017). A developmental cascade model of behavioral sleep problems and emotional and attentional self-regulation across early childhood. Behav Sleep Med.

[55] Becker K, Holtmann M, Laucht M, Schmidt MH (2004). Are regulatory problems in infancy precursors of later hyperkinetic symptoms?. Acta Paediatr.

[56] Polanczyk G, de Lima MS, Horta BL, Biederman J, Rohde LA (2007). The worldwide prevalence of ADHD: a systematic review and metaregression analysis. Am J Psychiatry.

[57] Oerbeck B, Overgaard KR, Aspenes ST, Pripp AH, Mordre M, Aase H, Reichborn-Kjennerud T, Zeiner P (2017). ADHD, comorbid disorders and psychosocial functioning: how representative is a child cohort study? Findings from a national patient registry. BMC Psychiatry.

[58] Atladottir HO, Parner ET, Schendel D, Dalsgaard S, Thomsen PH, Thorsen P (2007). Time trends in reported diagnoses of childhood neuropsychiatric disorders: a Danish cohort study. Arch Pediatr Adolesc Med.

[59] Thomas R, Sanders S, Doust J, Beller E, Glasziou P (2015). Prevalence of attention-deficit/hyperactivity disorder: a systematic review and meta-analysis. Pediatrics.

[60] Suren P, Bakken IJ, Aase H, Chin R, Gunnes N, Lie KK, Magnus P, Reichborn-Kjennerud T, Schjølberg S, Øyen A-S (2012). Autism spectrum disorder, ADHD, epilepsy, and cerebral palsy in Norwegian children. Pediatrics.

